# Effects of COVID-19 on Synaptic and Neuronal Degeneration

**DOI:** 10.3390/brainsci13010131

**Published:** 2023-01-12

**Authors:** Mohammed S. Alqahtani, Mohamed Abbas, Mohammad Y. Alshahrani, Khulud Alabdullh, Amjad Alqarni, Fawaz F. Alqahtani, Layal K. Jambi, Adnan Alkhayat

**Affiliations:** 1 Radiological Sciences Department, College of Applied Medical Sciences, King Khalid University, Abha 61421, Saudi Arabia; 2 BioImaging Unit, Space Research Centre, University of Leicester, Michael Atiyah Building, Leicester LE1 7RH, UK; 3 Electrical Engineering Department, College of Engineering, King Khalid University, Abha 61421, Saudi Arabia; 4 Electronics and Communications Department, College of Engineering, Delta University for Science and Technology, Gamasa 35712, Egypt; 5 Department of Clinical Laboratory Sciences, College of Applied Medical Sciences, King Khalid University, Abha 61421, Saudi Arabia; 6 Radiology Department, King Abdullah Hospital Bisha, Bisha 61922, Saudi Arabia; 7 Department of Radiological Sciences, College of Applied Medical Sciences, Najran University, Najran 55461, Saudi Arabia; 8 Radiological Sciences Department, College of Applied Medical Sciences, King Saud University, P.O. Box 10219, Riyadh 11433, Saudi Arabia; 9 Department of Hematopathology, King Fahad Central Hospital, Gizan 82666, Saudi Arabia

**Keywords:** COVID-19, brain atrophy, cerebrovascular diseases, CNS inflammatory diseases, peripheral neuropathy, demyelinating disorders, Parkinson’s disease

## Abstract

Neurons are the basic building blocks of the human body’s neurological system. Atrophy is defined by the disintegration of the connections between cells that enable them to communicate. Peripheral neuropathy and demyelinating disorders, as well as cerebrovascular illnesses and central nervous system (CNS) inflammatory diseases, have all been linked to brain damage, including Parkinson’s disease (PD). It turns out that these diseases have a direct impact on brain atrophy. However, it may take some time after the onset of one of these diseases for this atrophy to be clearly diagnosed. With the emergence of the Coronavirus disease 2019 (COVID-19) pandemic, there were several clinical observations of COVID-19 patients. Among those observations is that the virus can cause any of the diseases that can lead to brain atrophy. Here we shed light on the research that tracked the relationship of these diseases to the COVID-19 virus. The importance of this review is that it is the first to link the relationship between the Coronavirus and diseases that cause brain atrophy. It also indicates the indirect role of the virus in dystrophy.

## 1. Introduction

Cerebrovascular illness affects the brain’s blood flow and blood vessels. Blood flow issues may be caused by blood vessel constriction, clot development, artery blockage, or rupture. SARS-CoV-2 is an opportunistic brain infection that causes substantial respiratory distress, as well as neurological signs. COVID-19 is linked to symptoms including neurological complications and brain death.

**Figure 1:**
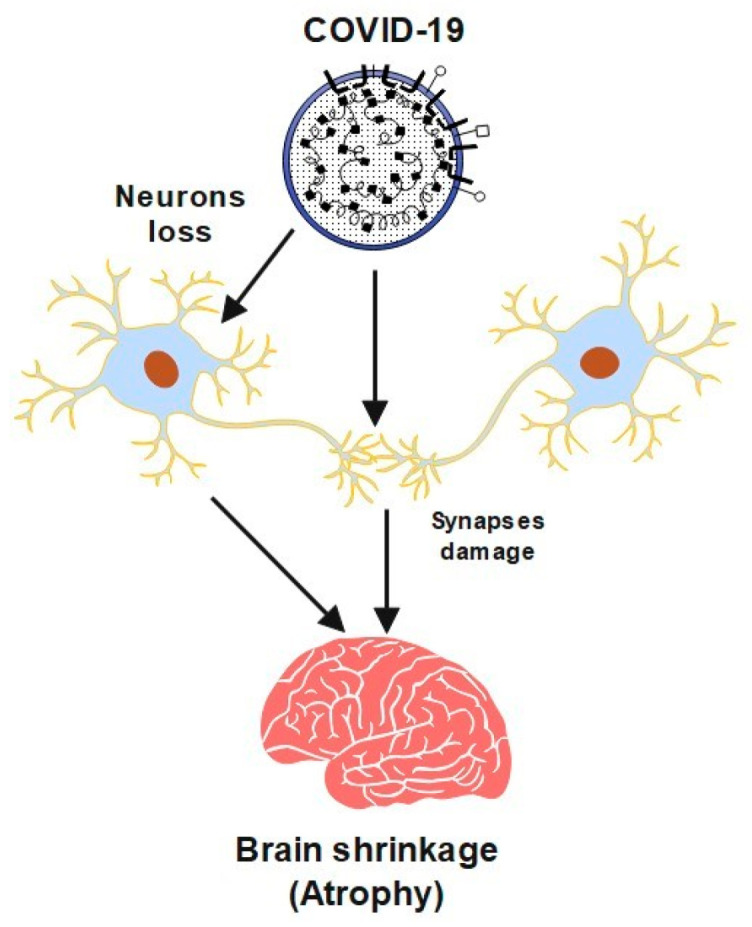
A schematic diagram of COVID-19 effects brain shrinkage.

However, studies have shown that this virus may cause neurological problems, which can sometimes precede usual symptoms like a fever and a cough. It may cause serious problems, including cerebrovascular disease (CVA), convulsions, or paralysis. The virus has caused serious neurological symptoms in older people and severely ill patients. Furthermore, COVID-19 can cause damage to neurons and their synapses. In this case, the brain could shrink, which leads to a brain disease called atrophy. A semantic diagram is presented in Figure 1 to show a summary of this process. It also has a significant influence on global mental health [1]. 

SARS-CoV-2 may trigger intrinsic and innate immunological responses in the host, including enhanced cytokine release, tissue damage, and high neurosusceptibility to COVID-19, particularly in hypoxic settings induced by lung injury. In immunocompromised people, the virus may enter the brain through the vasculature and peripheral nerves [2]. 

SARS-CoV-2 was detected in nasal swabs and a computerized tomography (CT) scan revealed widespread enlargement of the brain stem. A woman in her 59th year visited the emergency room after experiencing recurrent, fleeting episodes of vacant staring and speech arrest. These symptoms were accompanied by flexion of both shoulders and a brief generalized tonic-clonic seizure (GTCS), which was followed by postictal reduced consciousness. Due to her diminished state of awareness, she needed intubation and artificial ventilation.

It was found that the patient’s health had worsened with symmetrical hemorrhagic lesions in the brain stem, amygdalae, and thalamic nuclei. The findings matched hemorrhagic ANE with early brain stem involvement. The patient died after eight days of steroid treatment [3]. Postmortem brain magnetic resonance imaging (MRI) shows hemorrhagic and Posterior reversible encephalopathy syndrome (PRES)-related brain lesions in COVID-19 non-survivors, perhaps caused by virus-induced endothelium abnormalities and brainstem absence.

COVID-19 MRI abnormalities do not support brain-related respiratory distress [4]. SARS-CoV-2 may target deeper brain regions such as the thalamus and brainstem via trans-synaptic transmission, as seen in other viral illnesses. The virus may then infect the respiratory center of the brain, causing respiratory failure in COVID-19 patients. COVID-19 patients should be screened for neurological symptoms and the collapse of the respiratory center in the brainstem [5].

The pandemic’s neurological effects are becoming clearer, and COVID-19 does not seem to pass the blood-brain barrier [6]. The ubiquitous danger to our desire for human connection may combine with “brain styles,” which we previously characterized as “biotypes” informed by a neural taxonomy, to explain the mental health repercussions of COVID-19. This essay aims to promote research on COVID-19’s mental health effects from an individualized, brain-based approach that recognizes the virus’s grave danger to our core human drives [7].

Recent research shows that a cytokine storm promotes brain inflammation, and hence neurological symptoms during the COVID-19 epidemic. Targeting brain inflammation may help cure SARS-CoV-2’s neurologic consequences. Vascular Endothelial Growth Factor (VEGF), which is extensively distributed in the brain, may have a role in brain inflammation by promoting the recruitment of inflammatory cells and regulating angiopoietin II levels [8]. The vast variety of neurologic imaging results underscores the necessity for future investigations to improve therapy for these individuals [9]. The involvement of medulla oblongata brainstem structures in food intake and vomiting control has been studied, as well as the virus’s probable neurotropic and hematogenous routes to the brainstem [10].

Neurological exams are required for all SARS-CoV-2 patients, symptomatic or not, for COVID-19, and during hospitalization. Even after recovery from COVID-19, people should be considered at risk for neurological problems. Long-term neurologic monitoring following COVID-19 recovery will show whether this sickness is linked to treatable neurodegenerative illnesses. 

COVID-19 patients and their families should get mental health assistance when the condition has stabilized [11]. Many COVID-19 patients remain unconscious following a serious illness. We don’t know how structural brain abnormalities affect brain function or prognosis. This population has yet to be studied in terms of prognostic neuroimaging. Despite chronic non-responsiveness and anatomical brain abnormalities, a patient with severe COVID-19 showed intact functional network connections and, weeks later, regained command-following ability [12].

There is a link between anxiety and cognitive impairment in SARS-CoV-2-infected people with moderate or no respiratory symptoms and changed cerebral cortical thickness. Of all COVID-19 deaths, 19% had brain injuries. SARS-CoV-2 foci were found in astrocytes in all the afflicted brain regions. Neurotransmitter synthesis and energy consumption are altered in neural stem cell-derived astrocytes infected with a secretory phenotype that lowers neuronal survival [13].

Further study is required to understand the precise mechanisms and pathways of infectivity underpinning CNS pathology [14]. COVID-19 affects transcription in all cortical, parenchymal, and choroid plexus cell types. As a result of the SARS-CoV-2 infection of the cerebral vasculature and meninges, enhanced inflammatory signaling in the brain occurs. Parallel to this, peripheral immune cells penetrate the brain, microglia activate programs that mediate the phagocytosis of living neurons, and astrocytes dysregulate genes important in neurotransmitter balance. All these processes are part of the neuroinflammatory response [15]. COVID-19 symptoms may affect other organs, including the brain, and data on SARS-CoV-2 neuropathological characteristics is scarce [16].

**Figure 2:**
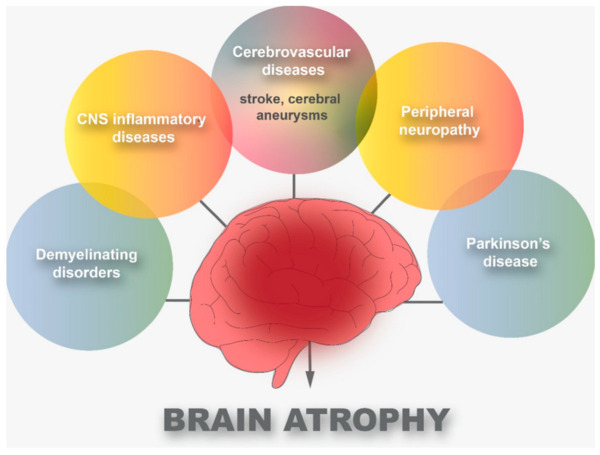
Common diseases that cause brain atrophy.

The effect of the COVID-19 quarantines on Traumatic brain injury (TBI) patients in Tyrol was investigated to ensure that neurosurgical treatment is available even during pandemic lockdown [17]. The COVID-19 infection can cause severe pneumonia, as well as systemic thrombotic complications, including cerebrovascular disease [18]. Infections of the CNS cause aseptic meningitis or encephalitis. The respiratory system is the major target of COVID-19. However, it is also a neuropathogen. The hallmark clinical feature ranges from mild confusion to profound coma. Most encephalitis patients are severely ill [19]. SARS-CoV-2 enters the brain through olfactory nerves via angiotensin-converting enzyme 2 (ACE2) and cytokine storms, a process called “cell reprograming” [20]. As shown in Figure 2, there are six main diseases that can cause brain atrophy: cerebrovascular diseases, CNS inflammatory diseases, peripheral neuropathy, demyelinating disorders, and PD. 

During the process of looking for references that were cited, we began with references that we had read in the past and that were relevant to the subject of the review. Following that, looking for other publications that have mentioned that reference is the next step. To put it another way, the reference that we began with was quite pertinent to our study, and other publications may have mentioned references that are also pertinent to our study. 

An alternative to searching with keywords has been utilized. When searching using cited references and searching with specific search phrases, such as the title and author of the referenced reference, you can find what you need. The main aim of this review is to shed light on the indirect role of the COVID-19 virus in brain atrophy. There are several diseases that lead to this atrophy in varying proportions from one disease to another and from one person to another, such as cerebrovascular diseases, CNS inflammatory diseases, peripheral neuropathy, demyelinating disorders, and Parkinson’s disease. However, recent research has been able to find a relationship between these diseases and COVID-19. This should help scientists understand the indirect role of COVID-19 in brain atrophy.

## 2. Cerebrovascular Diseases and COVID-19

Inhibitions of COVID-19 receptor ACE2 expression in a high-risk cohort for coronary heart disease (CHD) and stroke will be discussed next. A functioning ACE2 receptor has recently been identified in COVID-19, which may cause serious cerebrovascular disorders, including strokes, in people with risk factors for severe cerebrovascular diseases (CVD), such as diabetes and smoking. The effects of cigarette smoke extract (CSE) and hyperglycemia on ACE2 expression in arteries were studied in a rat MCAO model. After ischemic damage, the cortical penumbra expressed more ACE2. CSE increased ACE2 expression in the human brain vasculature. In diabetic primary cultured human blood vessels, ACE2 expression was increased [21]. Strokes have been reported in young people under fifty years old, with no cardiovascular risk factors associated with COVID-19. As of now, just a few instances have been reported, so it’s possible that the illness promotes its growth. Elderly people with stroke risk factors such as hypertension, diabetes, and increased fibrin D-dimers had higher cerebrovascular occurrences. COVID-19 contains several CVD cases reports and series. The mechanism that causes COVID-19 cerebral ischemia is unknown [22].

While the presence of both respiratory and cerebral injuries would suggest a greater severity, COVID-19 stroke patients might present with minor or non-respiratory symptoms. The severity of anterior circulation big vessel blockage strokes in COVID-19 patients was compared. Anterior circulation, big artery blockages and early brain imaging within 3 h of commencement were compared to a control group hospitalized over the same calendar month in 2019. Patients with COVID-19 had more severe big vessel occlusion strokes. The pandemic’s neurovascular effects should be feared given the enormous number of afflicted patients [23]. 

The pathogenetic effects of SARS-CoV-2 in the novel COVID-19 infection on the brain implies both direct and indirect harm. The goal of the research is to learn more about ischemic strokes in COVID-19 patients. Patients with COVID-19 need close monitoring of their coagulation system and aggressive thrombosis prevention. Providing ischemic stroke treatment in the context of COVID-19 adds to the organizational challenges, affecting intra-hospital logistics and care quality [24]. Stroke patients with COVID-19 had a 9-fold poorer prognosis than those without it [25]. SARS-CoV-2 induces COVID-19, a global condition. Swelling, cytokine storms, and an increase in heart injury biomarkers occur in the moderate and severe phases of infection. There may also be a link between COVID-19 and neurological problems. After being admitted to the Reanimation Unit, two patients with severe COVID-19 infection died. Moderate to severe COVID-19 patients should get high-dose antithrombotic prophylaxis [26].

COVID-19 seems to cause neurological complications, including bleeding and infarction. Individuals who are at high risk of coagulopathy should be investigated, but the risk of bleeding must be considered. Patients with COVID-19 should also have stringent blood pressure management. Acute cerebral infarction in COVID-19 individuals should be treated with thrombolytics [27].

According to reports, patients with COVID-19 have suffered from benign intracranial hypertension, seizures, ischemic and hemorrhagic cerebrovascular disorders, acute necrotizing encephalopathy, meningitis, and delirium. SARS-CoV-2 involvement in the central nervous system may worsen neurodegenerative problems in the future. The SARS-CoV-2 virus may harm the CNS. In turn, this leads to more severe symptoms and a higher chance of negative consequences [28]. SARS-CoV-2 has been shown to infect the CNS. The existing research on its neurological symptoms and pathological processes has not been systematic.

Patients may present with encephalopathy, encephalitis, seizures, cerebrovascular events, acute polyneuropathy, headache, hypogeusia, hyposmia, and other non-specific symptoms. However, they may indicate direct SARS-CoV-2-related neuronal injury [29]. COVID-19 has been linked to ischemic strokes. There is an increasing indication that COVID-19 effects extend beyond the lungs. Cerebrovascular disease is one of the most common neurologic symptoms [30]. 

The following aspects of cerebrovascular disease in COVID-19 patients will be discussed next: imaging, histology, and clinical features. There is a 1.4% incidence of cerebral vascular accident (CVA) in COVID-19 patients with substantial morbidity and death. Endotheliopathy with a haemorrhagic propensity has been linked to thrombotic microangiopathy [31]. SARS-CoV-2 infection may cause significant and multi-site vascular involvement. Results were classified as “relevant” or “other/incidental,” indicating the necessity for prompt patient care and treatment. The study groups were compared using the Student T-test, Mann-Whitney U-test, or Fisher exact test. Vascular involvement is not overlooked in COVID-19 and may have a substantial impact on disease behavior and prognosis [32].

The top protein of SARS-CoV-2 interacts with the ACE2 receptor. Twelve nucleotides are inserted into the S-polyphasic protein’s furin cleavage side at S1/S2 to slow down six receptor-binding domain (RBD) amino acids. Children with severe COVID-19 infections may benefit from angiotensin II receptor blockers [33]. 

Brainstem perivascular lymphocytic infiltration and microthrombi are common neurological consequences in COVID-19 patients, although little neuropathological research exists. A recent case report identified neocortical infarcts with tiny haemorrhagic and non-haemorrhagic white matter lesions, suggesting an evolving pattern of distinctive changes, which were also detected radiologically [34].

COVID-19 targets the lungs, causing an abrupt respiratory collapse. COVID-19 neurological symptoms include strokes, encephalitis, and neuropathy. The aspects of multiple sclerosis patient treatment during the COVID-19 epidemic are described [35]. Although SARS-CoV-2 usually affects the lungs, neurological symptoms are becoming more common. In three case studies of brain autopsies, no consistent pathobiological mechanism of CNS involvement in severe COVID-19 has been documented. Acute strokes, herniation, or olfactory bulb injuries were not seen [36].

Neuronal degeneration in COVID-19 individuals includes non-specific alterations in nerve cells. COVID-19 victims’ brain histopathology shows CNS impairment. Hypoxia from respiratory failure and individual risk factors including cerebrovascular atherosclerosis and hypertension induce ischemic strokes in COVID-19 patients [37]. SARS-CoV-2, which produced COVID-19, was recently found in postmortem neural and capillary endothelial cells of the frontal lobe of a patient. Seizures and early Guilli-an-Barre-like symptoms in SARS-CoV-2 patients have prompted concerns regarding the possible effects of SARS-CoV-2 neuroinvasion. The pathogenesis of SARS-CoV-2 infection is unknown [38]. Two COVID-19 patients had structural brainstem injuries. Two COVID-19-dead patients and two COVID-19-negative patients were studied for neuropathological characteristics. Histopathological, CA/mm^2^ and neuronal injury were measured. The medulla oblongata of COVID-19 patients had greater neuronal damage. SARS-CoV-2 in the brainstem and medullary damage in respiratory centers clearly imply a neurogenic component to COVID-19-related respiratory failure pathogenesis. This suggests viral trafficking between the brainstem and lungs [39]. 

## 3. Peripheral Neuropathy and CNS Inflammatory Diseases

### Peripheral Neuropathy and CNS Inflammatory Diseases

Several processes in the neurological system cause such manifestations. Virus-induced hyperinfammatory and hypercoagulable states are frequent examples. COVID-19 peripheral nervous system (PNS) diseases include Guillain-Barré syndrome (GBS) and its variations, sensory impairment (taste and smell), and muscular damage. CNS illnesses caused by COVID-19 include hemorrhagic and ischemic strokes, meningitis, encephalopathy, endotheliosis, and venous sinus thrombosis [40]. 

The diagnosis and treatment of cranial mononeuropathies, acute inflammatory Guillain-Barre polyneuritis, and severe brain and spinal cord damage caused by acute hemorrhagic necrotizing encephalopathy and myelopathies. SARS-CoV-2 has neurotropic and neurovirulence properties, and the combined impact of these variables, may produce neurological problems in COVID-19. COVID-19 influences chronic neurological illnesses, including neuroimmune diseases [41].

**Figure 3:**
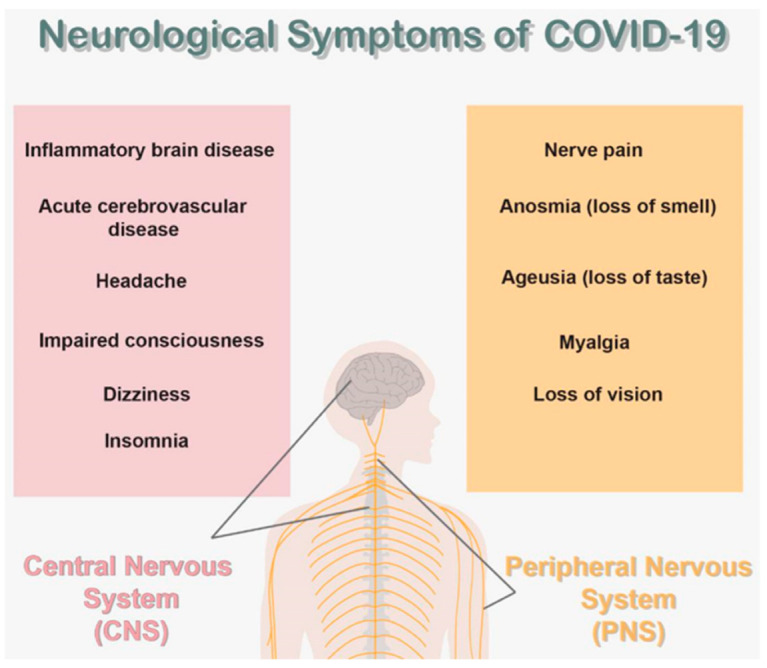
Neurological symptoms of COVID-19.

The Miller-Fischer variety of Guillain-Barré syndrome is an acute peripheral neuropathy caused by viruses, bacteria, and fungi. It causes ophthalmoplegia, ataxia, and areflexia. Miller-Fischer syndrome has recently been characterized in the clinical scenario of COVID-19 [42]. A 60-year-old man with SARS-CoV-2 infection, but only mild respiratory problems, developed akinetic mutism because of encephalitis. Electroencephalography revealed a global theta slowing. Interleukin-8 and tumor necrosis factor concentrations were raised in the acute stage of SARS-CoV-2 [43]. Figure 3 shows a schematic diagram of the main neurological symptoms of COVID-19.

Three months after the SARS-CoV-2 infection, up to 55% of COVID-19 hospitalized patients had neurological abnormalities. Its mutability and propensity to directly influence the CNS underlines the need to create technology to identify, manage, and treat COVID-19 patients’ brain injuries. The pathobiology of SARS-CoV-2 CNS infection, as well as its neurological consequences, remain unknown [44]. There have been four instances of COVID-19 encephalopathy. In COVID-19, individuals with new-onset cognitive problems, central focal neurological symptoms, or seizures were diagnosed. All patients had cognitive testing, MRI, and A fluorodeoxyglucose (FDG)-positron emission tomography (PET) / computerized tomography (CT) [45].

Human COVID-19 individuals show neurological abnormalities, both clinically and histologically. These symptoms may contribute to the disease’s morbidity and mortality, or perhaps signify the initiation of neurodegenerative activity in recovered individuals. COVID-19 enters cells via ACE2. SARS-CoV-2 may cause mild, moderate, or severe respiratory symptoms, leading to death. The viral receptor is found in the lungs, kidney, colon, and brain. Viral invasion causes cellular damage that leads to inflammation and decreases the activity of ACE2, which governs neuroprotective, neuro-immunomodulatory, and oxidative stress neutralizing capabilities. Hypoxemia occurs when patients are not given appropriate breathing assistance. The patient’s neurological symptoms will depend on characteristics such as age and sex, as well as the mechanism of neuronal invasion, the immunological response, and overall health [46].

Although the clinical signs are well understood, scientists are still unsure how SARS-CoV-2 enters and spreads throughout the CNS, causing brain injury. SARS-CoV-2 was recently found in Cerebrospinal Fluid (CSF) and frontal lobe slices from postmortem examinations, confirming its presence in neural tissue. In COVID-19 patients with underlying illnesses, this data points to a potential neurotherapeutic approach [47]. 

Coexisting microvascular alterations and upstream vascular tone all impact tissue oxygenation in critical organs. Infection and hypoxia-related inflammation impairs capillary function, which in turn increases hypoxia-related inflammation and tissue damage [48]. Initial structural neuroimaging investigations revealed widespread encephalopathies and damage to the medial temporal areas. Studies demonstrate that up to six months after hospitalization, over 30% of patients have cognitive deterioration and over 20% have serious mood control issues. Ongoing research in functional neuroimaging has confirmed anatomical results in frontal dysfunction. 

An electroencephalogram (EEG) investigation has shown higher deltas in frontal areas that match these results. In COVID-19, individuals with seizures, temporal, frontotemporal, and central-parietal focal zones have been discovered. Acute COVID-19 exposure and the neuroinflammatory effects of the COVID vaccine on non-exposed neurotypical individuals will be discussed. Current COVID-19 and future variant vaccines may stimulate the immune system, causing cytokine storms and neuroinflammatory consequences in certain people [49]. A 47-year-old man was taken to a hospital after suffering from worsening vertigo and ataxia for 7 days. The neurologic exam indicated cerebellar impairment, and the MRI showed cerebellar hemisphere edema with leptomeningeal enhancement. The Cerebrospinal fluid (CSF) indicated minor lymphocytic pleocytosis, protein, and lactate dehydrogenase elevations. CSF specimens had SARS-CoV-2 RNA. During hospitalization, the patients’ symptoms and signs improved significantly with lopinavir/ritonavir treatment [50]. Cerebral vasculitis-associated arterial vessel wall thickening was seen in 11 (16%) of the 69 COVID-19 patients. The difference between the 25 patients with and without SARS-CoV-2 infection was statistically significant (*p* = 0.03). The most often implicated vessels were the basilar and posterior cerebral arteries. Imaging revealed ischemia or hemorrhagic problems in nine. Cerebral vasculitis of medium-sized arteries seems to be one cause of COVID-19 brain injury [51]. 

Encephalopathy and encephalitis are severe SARS-CoV-2 CNS consequences. Hypoxic/metabolic alterations caused by the virus cause cytokine storms, acute respiratory distress syndrome (ARDS), and multi-organ failure. Hypoxic/metabolic encephalopathy is common among the elderly and seriously unwell. Comorbidities predispose patients to hypoxic/metabolic alterations that cause encephalopathy. These patients often need mechanical ventilation in the intensive care unit (ICU) [52]. 

## 4. COVID-19 and Parkinson’s Disease

### COVID-19 and Parkinson’s Disease

A higher incidence of serious neurological illnesses, such as encephalitis, is associated with COVID-19. Immunopathology and colonization of the intestine and central nervous system caused by SARS-CoV-2, as well as a systemic inflammatory response during COVID-19, may result in long-term autoimmune and neurological disorders. Neurodegenerative Parkinson’s disease involves both motor and non-motor symptoms [53]. The relationship between COVID-19 and PD is intriguing for many reasons. More than two decades ago, Coronavirus antibodies were discovered in the CSF of PD patients, suggesting a viral involvement in neurodegeneration [54].

As dopamine neurons have high ACE2 receptor expression and Parkinson’s disease has low ACE2 receptor expression due to degeneration, SARS-CoV-2–related brain penetration may cause additional injury, exacerbate symptoms, and increase the need for dopamine replacement therapy, as seen in 5 of the patients. As a result of Coronaviruses’ propensity to reach the brain via the nasal cavity, many infected people suffer from anosmia, hyposmia, and ageusia, which is a precursory sign of PD. As ACE2 and dopamine decarboxylase co-express and co-regulate in nonneuronal cell types, this may signal dopamine depletion and the need for levodopa therapy. The outcomes of SARS-CoV-2-infected PD patients are uncertain [54].

Unlike the 1918 influenza pandemic and the avian flu, COVID-19 has had few reports of encephalopathy and just one instance of Parkinson’s. Consensus guidelines for the clinical management of Parkinson’s patients with COVID-19 are suggested. However, specific motor and non-motor symptoms have been observed, and it will be crucial to follow participants after recovery, especially those with persistent hysteria [55]. 

COVID-19 has impacted everyone, particularly those with chronic conditions like Parkinson’s (PD). COVID-19 may influence PD patients’ motor and neuropsychiatric symptoms. COVID-19 was found in 18.18% of PD patients who had DBS.

There was no link between illness duration and COVID-19 prevalence. There was a statistically significant increase in COVID-19 prevalence in PD patients who had direct contact with SARS-CoV-2 infected people. There is no statistically significant link between deteriorating motor symptoms and COVID-19. Anxiety and sleep difficulties are common in PD patients, and COVID-19 may influence their psychological health. PD patients may have stricter preventative procedures, resulting in a lower prevalence and severity of COVID-19 and its effects. A more precise study is needed to determine if COVID-19 has any impact on the motor or psychosocial elements of PD [56].

Patients with deep brain stimulation face special hurdles because of the new Coronavirus illness (COVID-19) pandemic and public health initiatives to stop it using deep brain stimulation (DBS). During the countrywide lockdown from April to May 2020, patients with DBS had movement problems. There was an initial exacerbation of symptoms in two patients with subthalamic nucleus DBS for PD and one with globus pallidus interna DBS for generalized dystonia [57]. 

## 5. Fetal and Children Brain Damage

COVID-19 infection following SARS coronavirus 2 infection may cause significant pregnancy complications that affect neonatal outcomes. Physiological adaptations make pregnant women more vulnerable to cell-mediated viral infections. Pregnant women with COVID-19 are admitted to the An intensive care unit (ICU) at a slightly greater rate than non-pregnant women. Hypoxemia caused by maternal respiratory insufficiency may induce fetal distress. Amniotic fluid, fetal blood, chorionic villi, and fetal CSF samples were obtained for virological and genetic testing.

Atrophic cerebral cortex, dilated ventricles, hydrocephalus, and intraventricular hemorrhage were found at autopsy. Acute hypoxia induces changes in the whole CNS. Fetoplacental inflammation or microangiopathy were not detected [58]. Based on the COVID-19 pandemic, newborn SARS-CoV-2 infections are typically mild. There is weak data on how maternal infection impacts fetal development. An unusual instance of an intermediate-preterm child with cerebral hemorrhage and periventricular leukomalacia was reported [59].

The Healthy Brain and Child Development (HBCD) Study is an enormous longitudinal project that is co-funded by the Helping to End Addiction Long-Term (HEAL) Initiative of the National Institutes of Health, as well as several other institutes. Women from comparable environmental and socioeconomic backgrounds will be included in the HBCD sample, as will those with lower-risk profiles. The HBCD project will be able to analyze the developmental repercussions of neonatal medication withdrawal, which presently affects 8 out of every 1000 infants delivered in hospitals in the US. The HBCD sample will be racially and socioeconomically varied to capture a spectrum of developmental effects [60].

**Figure 4:**
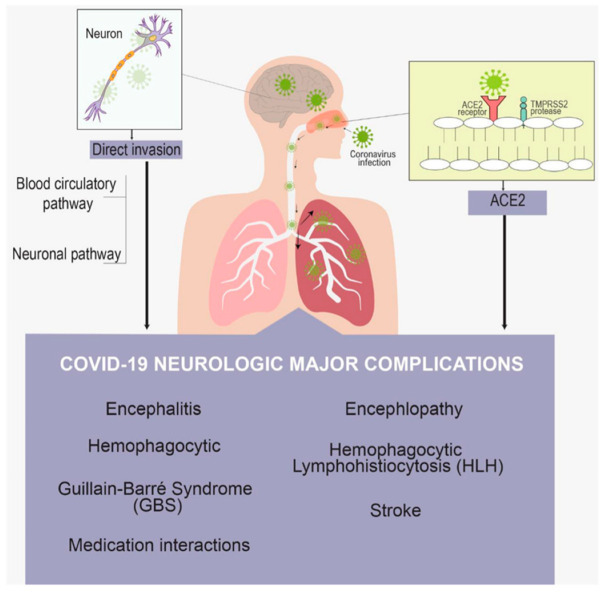
Neurologic major complications of COVID-19.

COVID-19 pandemic period pregnancies have been linked to poor fetal brain development and delayed cerebral cortical gyrification. Abnormal fetal brain development may have long-term neurodevelopmental consequences in COVID-era pregnancies compounded by increased mother anxiety [61]. All patients tested negative for N-methyl-d-aspartate receptor, Myelin oligodendrocyte glycoprotein (MOG), and aquaporin-4 autoantibodies. All three individuals had modest myopathic and neuropathic alterations on nerve conduction tests and electromyography. All patients showed neurological improvement, with two making a full recovery. On imaging, children with COVID-19 showed novel neurological symptoms involving the central and peripheral nervous systems but no respiratory symptoms [62]. Figure 4 shows the major neurologic complications of COVID-19.

Brain dysfunction may be caused by neuroplasticity in the nerves. The radiofrequency (RF) radiation from smartphones is unlikely to cause brain tumors in young people. The increased use may affect brain functioning, impair sleep and cognitive capacities, and increase the risk of mental diseases like depression, anxiety, Alzheimer’s disease, and ADHD [63]. Unusual brain development during pregnancy increases the likelihood of later mental illness. It is modeled in animals by maternal immune activation.

Like other known respiratory viruses, SARS-CoV-2 causes maternal inflammation during pregnancy. Choline, folic acid, vitamin D, and n-3 polyunsaturated fatty acids have all been researched as potential anti-infective and anti-inflammatory nutrients for pregnant women. The literature on their use in infected pregnant women is reviewed. Infants born to mothers who had viral infections in the first trimester benefit from higher maternal choline levels.

No other nutrient has been investigated in viral inflammation. Some research shows vitamin D decreases pro-inflammatory cytokines. Fluoride reduces anti-inflammatory cytokines. Unsaturated fatty acids have little impact [64]. Microglia, the brain’s resident immune cells, are influenced by the intrauterine environment, including maternal immune activity and inflammatory exposures. The COVID-19 pandemic presents a developmental immunological challenge to the embryonic brain, particularly when combined with maternal SARS-CoV-2 infection, which may result in cytokine storming. Therefore, they are unavailable throughout fetal life and after delivery; therefore, there is no existing biomarker or model for in utero microglial priming and function [65]. 

COVID-19 normally causes a moderate illness in children, although major consequences such as the multi-system inflammatory syndrome in children may arise (MIS-C). Adults have reported neurological symptoms ranging from moderate headaches to seizures, peripheral neuropathy, stroke, demyelinating diseases, and encephalopathy. The neurological consequences of COVID-19 appear to vary depending on age and underlying comorbidities [66].

The Behavior Assessment System for Children, Second Edition (BASC-2) The Parent Rating Scale was used to calculate connections between brain anatomy and internalizing and externalizing behavior. Internalizing behavior was linked to bilateral cingulum mean diffusivity (MD). In the cingulum, females had higher positive associations between MD and internalizing behavior. The left cingulum exhibited an age–behavior relationship, whereas the left uncinate fasciculus showed an age–behavior interaction. No behavior-brain volume associations survived multiple comparison corrections [67]. 

## 6. Alzheimer’s Disease (AD) and COVID-19

Defective brain connections are present in both Alzheimer’s patients [68,69].

The global proliferation of SARS-CoV-2 may have implications for Alzheimer’s disease. Neuroinflammation plays a role in Alzheimer’s etiology. The immunological response and increased inflammation caused by COVID-19 make elderly people more susceptible to SARS-CoV-2 infection. [70]. Many genetic risk variants have been linked to Alzheimer’s disease (AD). Anatomical models of the hippocampus, the primate dorsolateral prefrontal cortex (DLPFC), and lateral temporal lobe (LTL) These networks link AD SNPs to transcription factors and regulatory elements to uncover target genes linked to various AD symptoms. Machine learning techniques prioritize AD-COVID genes. The AD-COVID genes outperformed the known COVID-19 genes in predicting COVID severity and choosing patients for critical care [71].

Interpersonal interaction with COVID19 increases mortality in AD clinical trial participants. Clinical trials to prevent or treat Alzheimer’s disease still depend heavily on in-person cognitive testing. The Alzheimer’s Disease Assessment Scale-Cognitive (ADASCog) can be administered remotely with some adaptations and considerations. A novel technique to manage ADASCog’s virtual environment [72] Contrary to popular belief, professional tests frequently show neurological and cognitive issues. Seizures’ frequency, duration, and neurological cause are uncertain. These infections seem to harm the hippocampus, increasing the likelihood of memory loss and neurodegenerative diseases like Alzheimer’s [73].

Despite concerns regarding acceptable utilization by the elderly and the limits of evaluation without direct patient contact, the emergency phase gave an essential chance to examine telemedicine. Psychological and cognitive exams, as well as specialist professional figures, will be required to monitor and better comprehend the impacts of the ageing population [74]. The COVID-19 infection necessitates long-term neurological monitoring of COVID-19 patients, especially the elderly and sick. In this case, biomarkers can monitor COVID-19 patients for neurological problems like Alzheimer’s [75]. 

## 7. COVID-19 Patients with Anosmia 

Twenty-three individuals with COVID-19 olfactory dysfunction were assessed in [76]. A month elapsed between the beginning of the olfactory impairment and the examination. The Sniffin’ Sticks Test was used to assess olfactory function. We obtained olfactory nerve CTs and MRIs of the paranasal sinus. In MRIs, olfactory bulb volumes and sulcus depths were quantified, whereas morphology, signal intensity, and olfactory nerve filia architecture were assessed qualitatively. Based on olfactory tests, all patients were anosmic at the time of immaturity.

This was discovered on CT in 73.9 percent of patients, with mid- and posterior-segment dominance. Of the patients, 43.5 percent had small olfactory bulbs, and 60.9 percent had shallow sulci. In 54.2 percent of instances, the bulb’s usual inverted J shape changed. Ninety-three percent of patients showed diffusely elevated signal intensity, distributed hyperintense foci, or microhemorrhages. The olfactory bulb degradation rate was high. Further longitudinal imaging investigations may reveal the cause of olfactory neural circuit impairment [76].

Two additional participants with substantial odor abnormalities had mucosal hyperplasia of the upper nasal canals. Two participants with excellent scent restoration had no MRI anomalies. Olfactory system abnormalities may be the cause of anasmia in COVID-19 individuals [77].

Six individuals had bilateral olfactory cleft blockages, whereas three had minor olfactory bulb asymmetry. No MRI signal abnormalities were seen downstream of the olfactory tract. The core olfactory and high-order neocortical regions showed heterogeneous glucose metabolism problems. Using correlation analysis, we discovered that COVID-19-related dysosmia severity and duration affect regional cerebral glucose metabolism. The abrupt loss of smell in COVID-19 is not attributable to SARS-CoV-2 neuroinvasiveness.

Lack of olfactory input causes deafferentation and active functional reconfiguration in core olfactory and high-order cortical regions, resulting in metabolic alterations [78]. Identifying COVID-19 symptoms, particularly in the “asymptomatic” stage, and understanding the underlying molecular pathways will aid in establishing better prevention and treatment strategies. Given COVID-19’s expected second peak and its cyclical nature, taking action now would be prudent. Validated scoring tests for anosmia and dysgeusia is needed. Future research with large cohorts will help determine whether anosmia and dysgeusia are early COVID-19 indications that may assist in triaging confirmatory testing or implementing social distance and mask-wearing to limit community spread [79].

There is emerging evidence that COVID-19 patients have olfactory impairments. Anosmia may arise alone or in conjunction with other COVID-19 symptoms, including a dry cough. The underlying cause of olfactory impairment in COVID-19 individuals is unknown. Multiple cross-sectional investigations have shown that olfactory dysfunction occurs in 33.9–68 percent of COVID-19 patients, with a female predominance. COVID-19 patients often have anosmia and dysgeusia. Anosmia in outpatients should be noted by otolaryngologists to avoid the COVID-19 diagnosis [80].

## 8. Neurological Manifestation

Brain psychology and neurology both influence the brain. The stress caused by COVID-19 astounded and concerned medical experts and the general population. Headaches, sleeplessness, olfactory and gustatory impairments, and more severe encephalopathy were all associated with the COVID-19 neuroinvasion. The COVID-19 infection boosted immunity, resulting in severe lung and brain inflammation, with the former being the leading cause of COVID-19 death and the latter potentially leading to cerebral hemorrhage.

Hypercoagulation and aneurysm instability, which may be aggravated by systemic COVID-19 inflammation, may have a role in the development of mental illness. During the COVID-19 epidemic, people acquired behavioral patterns to cope with anxiety and stress, and they were more inclined to seek health information online, such as wearing face masks and washing their hands. To make the immune system more resistant to COVID-19, people were told to live a balanced life and get therapy [81].

Doctors and other healthcare practitioners may do regular motor function testing prior to an epidemic, monitor changes in cognition, mood, behavior, or quality of life, alter or titrate stimulation settings, and physically check battery life.

The bulk of these key therapeutic components are now accessible online, allowing patients to exercise more control and autonomy over their therapy. This substantial step forward in patient stimulation control is part of a broader attempt to establish a balance between battery conservation and symptom alleviation [82].

Allowing patients to “tweak” their therapy and experiment with different minimum thresholds within a safe range enables clinicians to “tweak” their treatment. Lockdown restriction measures have been associated with subjective worsening of motor and mental symptoms in patients undergoing deep brain stimulation for Parkinson’s disease or dystonia, implying that they may have exacerbated the burden of neurological illness and chronic stress associated with DBS treatment [83].

COVID-19 has been associated with delirium, acute and chronic attention, memory issues, and learning difficulties in adults and children because of hippocampal and cortical damage [84]. While the biology of COVID-19 infection and its long-term clinical outcomes are unknown, approaching the pandemic with lessons learned from previous infectious disease outbreaks and the biology of other coronaviruses will pave the way for the development of public mental health strategies that can be positively translated into therapeutic approaches aimed at improving stress coping responses, thereby aiding in pandemic alleviation [84].

COVID-19 has been linked to 123 distinct kinds of brain diseases, resulting in a complicated disease-disease network. By contrast, COVID-19 infection is more complex, with five closely packed modules, according to the brain-disease-gene network. The network’s hub proteins perform several functions, including catabolism of proteins, cell cycle regulation, RNA metabolism, and nuclear transport. 

Since drug repurposing affects these hub proteins, it improves therapy outcomes, such as comorbidity. According to doctors, people who test positive for COVID-19 are more likely to develop a range of additional neurological issues. To help minimize SARS-CoV-2’s long-term health implications, researchers must first understand the virus’s infection pathways and associated comorbidities [85].

Second, due to social isolation, many individuals who need mental health therapy are unable to get it. There is an urgent need for more accessible mental health treatment throughout the COVID-19 self-isolation period. Neurotherapeutics are increasingly available for home administration. Today, most in-home mental health treatment options are based on telehealth, which involves therapists communicating with patients through phone or video. 

Other treatments, such as self-administered, at-home brain stimulation, must be developed and made immediately available to aid in minimizing the negative mental health repercussions of self-isolation. One alternative is to employ existing therapeutic brain stimulation technologies for depression treatment on a home-based basis [86].

A growing collection of evidence suggests that the virus can infect the nervous system. Postmortem examinations have shown edema, hemorrhage, hydrocephalus, atrophy, encephalitis, infarcts, swollen axons, myelin loss, gliosis, neuronal satellitosis, hypoxic-ischemic damage, arteriolosclerosis, leptomeningeal inflammation, neuronal loss, and axon degeneration [87]. Additionally, the COVID-19 epidemic is wreaking havoc on global mental health, which may be explained in part by its cultural ramifications. Environmental variables may have contributed to the pandemic’s increased prevalence of psychiatric disorders, which are aggravated by psychological disorders. While performing clinical trials and identifying SARS-CoV-2-associated neurologic diseases are challenging challenges, they are crucial for understanding how COVID-19 manifests and burdens neurological and mental symptoms during and after the pandemic [87].

Numerous underlying autoantigens and their molecular similarities to SARS-CoV-2 are unknown. Autoantibodies may now be utilized to explain some aspects of COVID-19’s multiorgan sickness and to guide treatment in a subset of patients [88]. Virus infections have a substantial impact on brain functions and have the potential to cause serious neurological damage. Coronaviruses (CoV), most notably SARS-CoV-2, have been demonstrated to have neurotropic characteristics and the ability to cause neurological disorders. CoV was discovered in the brain and CSF fluid, according to research. Due to the unknown pathobiology of these neuroinvasive viruses, study into the neurological repercussions of CoV infection is crucial [89].

The COVID-19 news reporting variance was associated with a variety of behaviors, including increased phone usage, decreased physical activity, and fewer online visits. While widespread changes in mental health and behavior are to be expected, recognizing them is crucial for establishing strategies aimed at mitigating the mental health repercussions of future disasters [90].

The COVID-19 infection is neurologically like the SARS-CoV-2 illness in terms of neurological symptoms. Despite its propensity to infect the neurological system directly, SARS-CoV-2 has been found in the CSF just twice [91]. To explain the clinical relevance of SARS-neuropathogenicity CoV-2s and to assess the potential impact of SARS-CoV-2 infection on common neurological illnesses, globally standardized registries are required [91]. Individuals with COVID-19 often have neurological difficulties.

When treating patients with neurologic symptoms during the COVID-19 pandemic, doctors should consider coronavirus infection as a differential diagnosis to avoid unnecessary delays or misdiagnosis, and to avoid missing an opportunity to cure and prevent future transmission [92]. COVID-19 is a virus that has the potential to infect the central and peripheral nervous systems, as well as other organs. In people of all ages, COV generates similar neurologic symptoms, with headaches (16.8 percent), dizziness (13.9 percent), and altered awareness being the most prevalent (13.9 percent).

The virus’s neurotropic potential is demonstrated in 11.2 percent of CoV infections of the CNS through the production of numerous cytokines and possibly immune system failure [93]. Physicians and other healthcare providers may notice COVID-19 neurologic signs in patients who are at high risk. COVID-19 may be aimed at the neurological system because of the higher occurrence of olfactory and taste disorders, myalgia, headaches, and acute cerebral vascular disease. Two of the most prevalent entry sites into the neurological system are cytokine production and blood circulation [94].

According to the current study, COVID-19 patients may have neurologic symptoms before, during, or even after the onset of typical COVID-19 symptoms. Dizziness, headaches, myalgia, weariness, altered awareness, disorientation, ageusia, anosmia, neuropathic or radicular pain, occipital neuralgia, visual impairment, seizures, and ataxia are only a few of the most frequently encountered neurological symptoms and signs. COVID-19 may cause GBS, myopathy, rhabdomyolysis, and acute myelitis by damaging the CNS, PNS, and skeletal muscles, according to an increasing number of case reports and research. The actual cause of these issues is unknown. The nervous system communicates directly with itself. Infection with SARS-CoV-2, neuroinflammation, a post-viral immunological response, hypercoagulability, and metabolic or hypoxic damage have all been suggested as possible causes [95].

Even though other betacoronaviruses have comparable characteristics, only a few studies have shown SARS-CoV-2 brain infection in vivo. A neuronal and hematogenous bridging of the blood–brain barrier has been postulated considering recent discoveries that SARS-CoV-2 host cell entrance receptors exist as components of human neurons and vascular tissue. COVID-19 neurological symptoms may be a consequence of both the neuroinflammation generated by the “cytokine storm” and coexisting diseases.

Additionally, SARS-CoV-2 may reach the brain through a variety of different channels, or it may be implicated in the development of neurological symptoms via a variety of different processes. [96]. The analysis excluded patients with missing or non-contributory brain MRI data, ischemic infarctions, cerebral venous thrombosis, or chronic abnormalities unrelated to the present incidence. To compare categorical data, the Fisher exact test was utilized. Patients with elevated COVID-19 levels, but no evidence of ischemia infarction, had a variety of neurologic symptoms, as well as an abnormal brain MRI [97]. There have been eight distinct neuroradiological patterns.

The researchers used fractional anisotropy (FA), mean diffusivity (MD),axial diffusivity (AD), and radial diffusivity (RD), as well as an index grading system, to grade diffusion tensor imaging (DTI). We compared regional volumes estimated using voxel-based morphometry (VBM) to DTI data using covariance analysis (ANCOVA). The correlations between imaging indices, index scores, and clinical data were determined using a two-sample *t*-test and Spearman correlation. Findings at this point in the trial show 55% of COVID-19 participants reported neurological symptoms [98].

The most common neuroimaging findings are acute infarcts with a high clot load and cerebral bleeding, including microhemorrhages. Each of these imaging patterns is possible in leukoencephalopathy, global hypoxia, acute demyelinating encephalomyelitis, corpus callosum cytotoxic lesions, olfactory bulb involvement, cranial nerve enhancement, and Guillain-Barré syndrome. Immunological activation is the most common cause of central nervous system disease, since it results in a prothrombotic state and a cytokine storm; direct neuro-invasion is unusual [99]. A growing collection of evidence suggests that the virus can infect the nervous system.

Postmortem examinations have shown edema, hemorrhage, hydrocephalus, atrophy, encephalitis, infarcts, swollen axons, myelin loss, gliosis, neuronal satellitosis, hypoxic-ischemic damage, arteriolosclerosis, leptomeningeal inflammation, neuronal loss, and axon degeneration. While clinical exams to detect neurological issues associated with SARS-CoV-2 are tough, they are necessary to have a better knowledge of COVID-19’s presentation and burden of neurological and mental symptoms during and after the pandemic [100]. 

COVID-19 patients with recovered frontal gray matter volumes, increased white matter hyperintensities, and decreased MD in total, and normal-appearing white matter in the posterior thalamic radiation and sagittal stratum had decreased MD in total and normal-appearing white matter compared to controls. These alterations were more pronounced in recipients of organ transplants. 

The T2* levels in the thalamus were shown to be increased in recovered COVID-19 patients, with the rise being greatest in non-critical patients [101]. COVID-19, or its concurrent prescription, has been linked to a specific pattern of microbleeds in the brain, most notably in the corpus callosum, according to brain MRI. Thrombotic microangiopathy caused by SARS-CoV-2 infection, as well as hypoxemia-induced collapse of the brain blood barrier, should be investigated [102]. Vascular injury, particularly to microvessels, is theorized to occur. The underlying mechanisms, which include viral infection of the central nervous system and systemic cardiovascular problems, are unclear, and there is no evidence of a causal relationship with SARS-CoV-2 infection [103].

## 9. Conclusions and Future Perspectives

In the human body’s neurological system, neuronal cells serve as the fundamental building blocks. When cell-to-cell contact is disrupted, the affected area loses its capacity to function. In addition to peripheral neuropathy and demyelinating illnesses, there is a substantial link between Parkinson’s disease and several other ailments. An inverse relationship has been shown between these conditions and the shrinking of the brain. After a period of infection with one of these illnesses, it may take some time before the atrophy is detected.

The virus can cause any of the disorders that were previously linked to brain shrinkage in research. The findings of our study will allow us to have a better understanding of the relationship between these illnesses and the COVID-19 virus. In this review, it was discovered that there was a link between the coronavirus and illnesses that cause brain shrinkage for the first time. It also seems to show that the virus plays a less direct role in the development of muscular dystrophy.

In the future, meta-analysis will be carried out, and additional research should be investigated to justify the reason for the spread of the virus and possible preventative measures. As a result, additional efforts should be made to collect and organize all the information available on the COVID-19 disease from its various sources. A thorough look at the data from retrospective cohort studies and a discussion of where COVID-19 came from, its pathophysiology, epidemiology, and possible entry points for SARS-CoV-2 into the brain are also part of the report. 

## Data Availability

The data presented in this study are available within the article.
